# Melatonin controls microbiota in colitis by goblet cell differentiation and antimicrobial peptide production through Toll-like receptor 4 signalling

**DOI:** 10.1038/s41598-020-59314-7

**Published:** 2020-02-10

**Authors:** Seung Won Kim, Soochan Kim, Mijeong Son, Jae Hee Cheon, Young Sook Park

**Affiliations:** 1 Department of Internal Medicine and Institute of Gastroenterology, Yonsei University College of Medicine, Seoul, Korea; 2 Brain Korea 21 PLUS Project for Medical Science, Yonsei University College of Medicine, Seoul, Korea; 3 Severance Biomedical Science Institute, Yonsei University College of Medicine, Seoul, Korea; 4 Department of Internal Medicine, Eulji Hospital, Eulji University School of Medicine, Seoul, Korea

**Keywords:** Dysbiosis, Inflammatory bowel disease

## Abstract

Microbial dysbiosis has long been postulated to be associated with the pathogenesis of inflammatory bowel disease (IBD). Although evidence supporting the anti-colitic effects of melatonin have been accumulating, it is not clear how melatonin affects the microbiota. Herein, we investigated the effects of melatonin on the microbiome in colitis and identified involvement of Toll-like receptor (TLR) 4 signalling in the effects. Melatonin improved dextran sulfate sodium (DSS)-induced colitis and reverted microbial dysbiosis in wild-type (WT) mice but not in TLR4 knockout (KO) mice. Induction of goblet cells was observed with melatonin administration, which was accompanied by suppression of *Il1b* and *Il17a* and induction of melatonin receptor and Reg3β, an antimicrobial peptide (AMP) against Gram-negative bacteria. *In vitro*, melatonin treatment of HT-29 intestinal epithelial cells promotes mucin and wound healing and inhibits growth of *Escherichia coli*. Herein, we showed that melatonin significantly increases goblet cells, Reg3β, and the ratio of *Firmicutes* to *Bacteriodetes* by suppressing Gram-negative bacteria through TLR4 signalling. Our study suggests that sensing of bacteria through TLR4 and regulation of bacteria through altered goblet cells and AMPs is involved in the anti-colitic effects of melatonin. Melatonin may have use in therapeutics for IBD.

## Introduction

Inflammatory bowel disease (IBD) is a chronic relapsing disorder of the gastrointestinal (GI) tract^1^ that results in chronic diarrhoea, abdominal pain, weight loss, and altered gastrointestinal motility^2^. The pathogenesis of IBD is considered to be multidimensional and multifactorial, involving genetic and environmental factors. The microbiota (microbial community assemblage) plays a critical role in the GI tract and is involved in nutrient digestion, vitamin synthesis, and promotion of mucosal immune tolerance. Perturbation of the intestinal microbiota composition and inflammatory response can aggravate intestinal barrier dysfunction and is a risk factor for IBD, although the mechanisms of IBD pathogenesis remain unclear. While various pharmaceutical treatments are available for IBD^3^, some therapeutic agents have a higher risk of toxicity and infectious complications^4^. Recently, microbiota control using faecal microbiota transplantation, probiotics, and prebiotics has been proposed as a new therapeutic option^5^.

Melatonin (N-acetyl-5-methoxy^5^ tryptamine) is a pineal gland hormone that regulates other hormones, circadian rhythm, and oxidative stress^6^. Recent experiments have shown that melatonin also acts as an immune system modulator^7^ and ameliorates DSS colitis in mice^8,9^. Numerous studies have indicated that melatonin affects the permeability, motility, and barrier function of the intestine^10,11^. Melatonin treatment can improve GI tract diseases, such as irritable bowel syndrome^12^, IBD^13^, and necrotizing enterocolitis^14^, suggesting it has a critical role in the physiologic function of the GI tract. Interestingly, melatonin accumulates in the GI tract independent of production from the pineal gland. The concentration of melatonin in the GI tract is 400-fold greater than that in the pineal gland^15^. The GI tract is also the largest source of enzymes for melatonin production^16^, and melatonin receptors are highly expressed in the GI tract, suggesting that melatonin affects the immune response and the microbiota. Recent studies reported that melatonin increases the ratio of *Firmicutes* to *Bacteroidetes* and *Akkermania*^10^ and suppresses pathogenic bacteria^17^ in the intestine. Despite this knowledge, the pivotal signal pathway for the anti-colitic effect of melatonin and the exact mechanism for control of the intestinal microbiota remains unknown.

We previously reported that melatonin improves sleep deprivation-induced colitis in mice^13^. In this study, we investigate the involvement of Toll-like receptor (TLR) 4 signal in the action of melatonin. TLR4 is an important TLR that recognizes pathogen-associated molecular patterns^18^, especially Gram-negative bacteria, and controls intestinal epithelial cells and the mucosal barrier. We also explored the effects on microbiota dysbiosis and antimicrobial peptide (AMP) regulation of melatonin in colitis.

## Results

### Intraperitoneal administration of melatonin ameliorates DSS-induced colitis through TLR4 signalling

**Figure 1:**
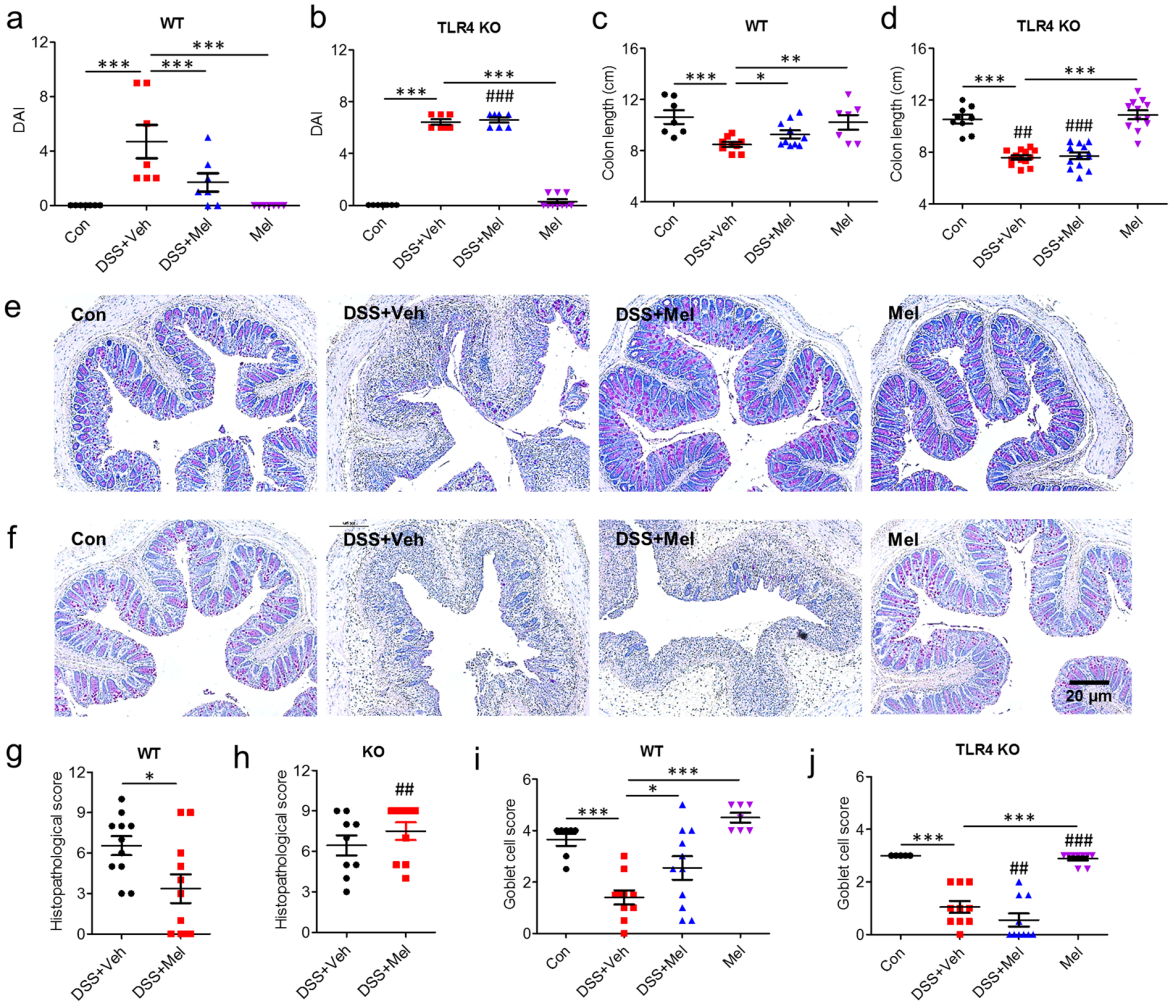
Intraperitoneal administration of melatonin ameliorates DSS-induced colitis through TLR4 signal pathway. Wild type (WT) and TLR4 knockout (TLR4 KO) mice were administered 2.5% DSS in drinking water and treated i.p. with 0.25% EtOH/PBS (Veh) or melatonin (Mel, 10 mg/kg/day) from days 1 to 8. (**a**,**b**) Disease activity index (DAI). (**c**,**d**) Colon length. (**e–j**) Histopathology of colon. (**e**,**f**) Representative image of periodic acid-Schiff (PAS)-stain. (**g**,**h**) Histopathologic score. (**i**,**j**) Goblet cell score. Data represent mean ± S.E.M. (n = 10). Statistical significance was assessed using one-way ANOVA followed by Dunnett post-test compared to DSS + Veh. *P < 0.05, **P < 0.01, ***P < 0.005, ^##^*P* < 0.01 vs. TLR4 KO, ^###^*P* < 0.005 vs. TLR4 KO. Con, untreated control.

TLR responses in intestinal epithelium by commensal bacteria play a significant role in the innate immunity, colonic homeostasis, and tolerance that link luminal bacteria and intestinal inflammation^19^. Among these TLRs, TLR4 recognize lipopolysaccharide (LPS) in particular and is primarily involved in control of Gram-negative bacteria; an association between genetic polymorphisms of TLR4 and IBD have been reported, although some controversy remains^19^. TLR4 knockout (KO) mice are highly susceptible to Gram-negative bacteria^20,21^. Thus, to explore the involvement of TLR4 signalling in the anti-colitic effects of melatonin *in vivo*, colitis was induced in 8-week-old wild type (WT) and TLR4 KO mice by administration of 2.5% dextran sulfate sodium (DSS) in drinking water and intraperitoneal (i.p.) treatment with melatonin (Mel) or vehicle (Veh). Body weight changes were monitored daily. DSS-treatment for 10 days significantly increased body weight loss in the vehicle-treated group of both wild type and TLR4 KO mice. Although there were no significant differences in body weights between DSS + Veh and DSS + Mel groups (Fig. S1a,b), melatonin improved the disease activity index (DAI) (Fig. 1a,b) and attenuated shortening of the colon (Fig. 1c,d) only in wild type mice but not in TLR4 KO mice.

### Defects of TLR4 signalling abolishes induction of goblet cells and anti-inflammatory response caused by melatonin

Colons of the DSS-treated group indicated severe inflammation with inflammatory cell infiltration and microscopic damage including transmural injury, crypt and epithelium loss, and goblet cell loss. In contrast, the melatonin-treated group of wild type mice showed significant improvement in histopathologic features compared to the vehicle-treated group, while these effects were not observed in TLR4 KO mice (Fig. 1e–h). Of note, periodic acid-Schiff (PAS) staining indicated that the melatonin-treated control group displayed drastically increased goblet cells in colons, whereas TLR4 KO mice showed a trend toward decreased goblet cells compared to WT mice (Fig. 1e,f,i,j).

**Figure 2:**
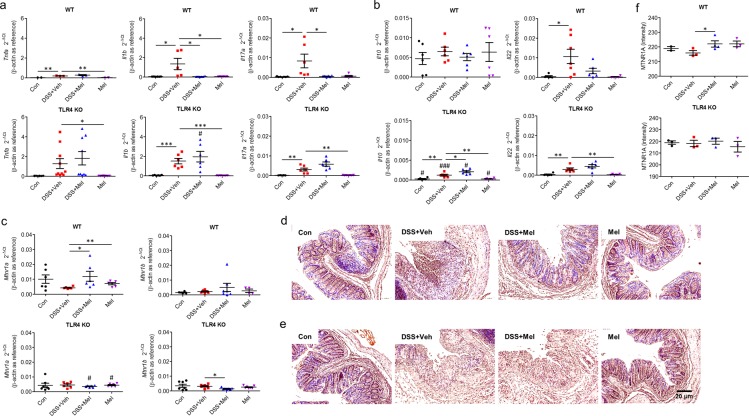
Melatonin modulates inflammatory cytokines and melatonin receptors in the colon through TLR4 signal pathway. (**a–c**) Gene expression in colon tissues. mRNA expression of pro-inflammatory cytokines (*Tnfa*, *Il1b*, *Il17a*) (**a**), anti-inflammatory cytokines (*Il10*, *Il22*) (**b**), and melatonin receptors (*Mtnr1a*, *Mtnr1b*) (**c**) were evaluated by quantitative RT-PCR. (**d–f**) Protein levels of melatonin receptors in colon tissues from WT (**d**) and TLR4 KO (**e**) mice. (**d**,**e**) Representative image of immunohistochemistry for melatonin receptor 1 A (MTNR1A). (**f**) Densitometric analysis. Data represent mean ± S.E.M. (n = 7). Statistical significance was assessed using Student’s t-test. *P < 0.05, **P < 0.01, ***P < 0.005, ^#^*P* < 0.05 vs. TLR4 KO, ^###^*P* < 0.005 vs. TLR4 KO. Con, untreated control; Mel, melatonin-treated; TLR4 KO, TLR4 knockout mice; Veh, vehicle-treated; WT, wild-type mice.

Colitis is characterized by expression of both pro-inflammatory cytokines, including tumour necrosis factor-alpha (*Tnfa*), interleukin 1-β (*Il1b*), and interleukin 17 A (*Il17a*), and anti-inflammatory cytokines, including interleukin 10 (*Il10*) and interleukin 22 (*Il22*). Gene expression of these cytokines in colons was investigated. Real-time quantitative reverse-transcription polymerase chain reaction (qRT-PCR) analysis indicated that melatonin significantly suppressed induction of *Il1b* and *Il17a* in WT mice, which was not true in TLR4 KO mice (Fig. 2a). Of note, TLR4 KO mice showed a trend of increased *Tnfa*, *Il1b*, and *Il17a* but reduced *Il10* compared to WT mice (Fig. 2a,b). Taken together, these results indicate that melatonin modulates goblet cell differentiation and expression of inflammatory cytokines via TLR4 signalling.

### Melatonin alter microbiota composition and corrects gut dysbiosis

There are two different receptors for melatonin, type 1 A (MT1) and type 1B (MT2), which are encoded by *Mtnr1a* and *Mtnr1b*, respectively^22^ and expressed in the intestine^23–25^. Expression of melatonin receptors (*Mtnr1a* and *Mtnr1b*) was assessed to investigate changes in melatonin and TLR4 signalling. qRT-PCR showed significantly increased expression of *Mtnr1a* and an increasing trend of *Mtnr1b* in colons of melatonin-administered mice compared to vehicle-treated groups when also treated with DSS (Fig. 2c). However, expression levels of *Mtnr1a* showed a decreased trend in TLR4 KO mice compared to WT mice. Immunohistochemistry staining for MT1 in colon sections also supported these results (Fig. 2d–f). TLR4 is an important signal pathway in homeostasis between hosts and pathogens. The results indicate that melatonin signalling is linked to TLR4 signalling and affects the microbiota, and there is a critical modulator dependent on TLR4 signalling.

**Figure 3:**
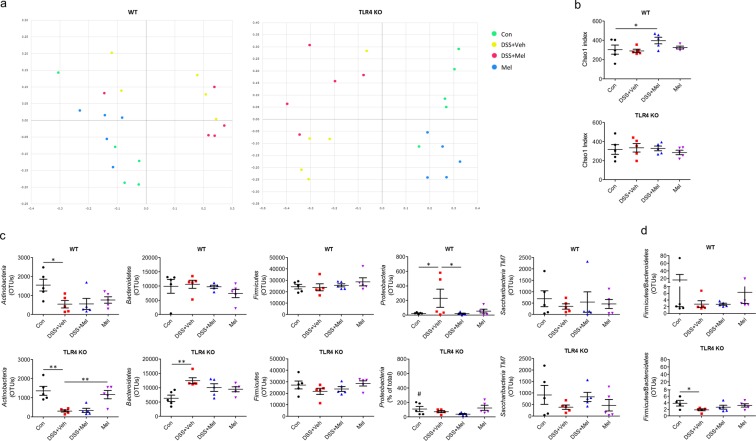
Melatonin suppresses dysbiosis of gut microbiome through TLR4 signal pathway. Fecal microbiome composition was generated using 16 S rRNA sequencing, and beta and alpha diversity and taxa were analysed. (**a**) Principal coordinate analysis (PCoA). (**b**) Chao1 index. (**c**) Microbiota profiles in phylum level. (**d**) *Firmicutes* to *Bacteroidetes* ratio. Data represent mean ± S.E.M. (n = 5). Statistical significance was assessed using Student’s t-test (**b**,**d**) and one-way ANOVA followed by Dunnett post-test (**c**). *P < 0.05, **P < 0.01. Con, untreated control; Mel, melatonin-treated; OUT, operational taxonomic unit; TLR4 KO, TLR4 knockout mice; Veh, vehicle-treated; WT, wild-type mice.

First, to observe changes in microbiota caused by melatonin, metagenome analysis was performed using faeces of WT and TLR4 KO mice. 16 S rRNA gene high-throughput pyrosequencing revealed the overall microbiome composition (beta diversity) assessed using principal coordinate analysis (PCoA), which showed distinct differences in clustering between the microbiomes of vehicle- and melatonin-treated groups and between WT and TLR4 KO mice (Fig. 3a). In alpha-diversity analysis, the melatonin-administered group showed significantly increased richness of faecal microbiota assessed using Chao index (Fig. 3b), increasing trends in ACE and Shannon diversity index, and a decreased trend of Simpson index compared to DSS + Veh groups (Fig. S2a), indicating that melatonin increased richness and diversity of the microbiome. Of note, melatonin-treated groups showed significant suppression of *Proteobacteria*, a representative Gram-negative phylum that includes *Salmonella* and *Escherichi coli* (Fig. 3c), and increased *Ruminococcaceae* family strains (Fig. S2d), which represent butyrate-producing Gram-positive bacteria decreased in abundance in IBD fecal microbiota^26,27^. While the difference was not significant, melatonin-treated groups showed a decreasing trend in *Bacteroidetes* and an increasing trend of *Firmicutes* relative abundance compared to vehicle-treated groups (Fig. 3c) as previous studies^17,28^. A significantly decreased *Firmicutes* to *Bacteroidetes* ratio in DSS-treated groups was also shown in TLR4 KO mice, whereas a significantly decreased abundance of *Proteobacteria* was shown in DSS-treated groups of WT mice (Figs. 3c,d and S2b–f).

### Melatonin controls Gram-negative bacteria through induction of Reg3β

**Figure 4:**
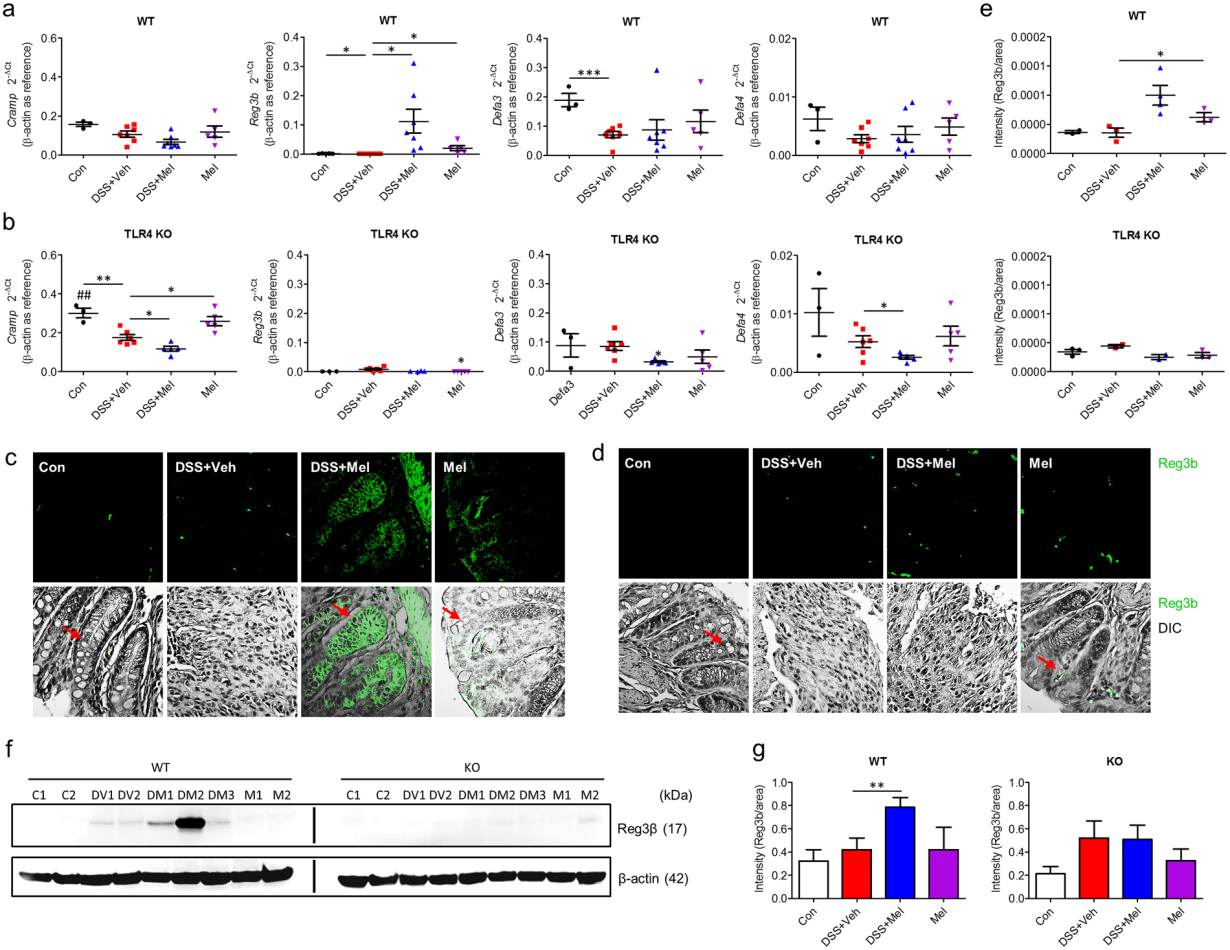
Melatonin promotes production of antimicrobial peptides through TLR4 signal pathway. (**a**,**b**) Gene expression of antimicrobial peptides in colon tissues. mRNA levels of *Cramp*, *Reg3b*, *Defa3*, and *Defa4* in WT (**a**) and TLR4 KO (**b**) mice were evaluated by quantitative RT-PCR. (**c**–**e**) Protein levels of Reg3β in colon tissues from WT and TLR4 KO mice. (**c**,**d**) Representative image of immunofluorescence stain for Reg3β in colons of WT (**c**) and TLR4 KO (**d**) mice. Red arrows indicate representative goblet cells. (**e**) Densitometric analysis of immunofluorescence stain. Data represent mean ± S.E.M. (n = 7). (**f**) Representative image of western blot for Reg3β in colon lysates of WT and TLR4 KO mice. (**e**) Densitometric analysis of western blot. Statistical significance was assessed using Student’s t-test. *P < 0.05, **P < 0.01, ***P < 0.005, ^##^*P* < 0.01 vs. TLR4 KO. Con or C, untreated control; DSS or D, DSS-treated; Mel or M, melatonin-treated; TLR4 KO, TLR4 knockout mice; Veh or V, vehicle-treated; WT, wild-type mice.

Next, gene expression related to AMPs in the proximal colon was examined, including cathelin-related antimicrobial peptide (*Cramp*), regenerating islet-derived protein 3 beta (*Reg3b*), defensin alpha 3 (*Defa3*), and defensin alpha 4 (*Defa4*), to explore the mechanism of microbiota regulation in the colon. Among these AMPs, only *Reg3b* expression was significantly increased in melatonin-administered groups compared to other groups, in which *Reg3b* expression was undetectable. Expression of other intestinal antimicrobial molecules was not affected (Fig. 4a,b). In concordance, protein levels assessed by immunostaining and western blot were also increased in the melatonin-administered groups (Fig. 4c–g). However, TLR4 KO mice showed reduced levels of inducible AMPs, such as Reg3β and defensins. This suggests that melatonin induces expression of AMPs, in particular Reg3β, through TLR4 signalling.

**Figure 5:**
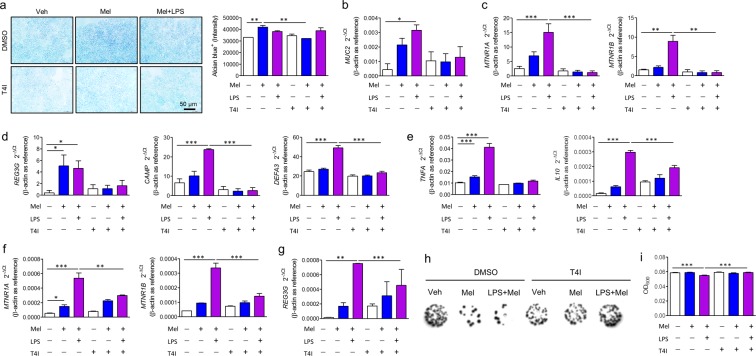
Melatonin controls Gram-negative bacteria by goblet cell differentiation and antimicrobial peptide production through TLR4 signal pathway. (**a**–**d**) HT-29 cells were treated with vehicle (Veh) or melatonin (Mel) with or without TLR4 inhibitor (T4I) for 48 h. (**a**) Representative image of Alcian blue stain (left) and densitometry analysis (right). (**b**–**d**) Gene expressions of melatonin receptor, mucin, and antimicrobial peptides in intestinal epithelial cells (IECs). mRNA levels of mucin 2 (*MUC2*) (**b**), melatonin receptor (*MTNR1A*, *MTNR1B*) (**c**), and antimicrobial peptides (*REG3B*, *CAMP*, *DEFA3*) (**d**) were evaluated by quantitative RT-PCR. Data represent means ± S.E.M. of 3 independent experiments. (**e–g**) The differentiated THP-1 cells were treated with vehicle or melatonin with or without TLR4 inhibitor for 4 h. mRNA levels of cytokines (*TNFA*, *IL10*) (**e**), melatonin receptor (*MTNR1A*, *MTNR1B*) (**f**), and antimicrobial peptides (*REG3B*) (**g**) were evaluated by quantitative RT-PCR. (**h**,**i**) Antimicrobial activity of melatonin-treated cells. *Escherichia coli* (OD_600_ = 0.5) grown in LB broth were treated with culture media from HT-29 cells, incubated for 48 h, diluted at 1/10^5^, and seeded on agar plate. (**h**) Representative image of colonies on agar plate. Diluted bacterial cultures in the presence of HT-29 culture media were spot-inoculated onto EMB agar plate and captured in Gel document system. (**i**) Optical density (OD_600_) of *E. coli* culture. Data represent mean ± S.E.M. of 4 independent experiments. Statistical significance was assessed using one-way ANOVA followed by Dunnett post-test. *P < 0.05, **P < 0.01, ***P < 0.005, ^#^*P* < 0.05 vs. DMSO, ^##^*P* < 0.01 vs. DMSO, ^###^*P* < 0.005 vs. DMSO. DMSO, dimethyl sulfoxide-treated; L*P*S, lipopolysaccharide-treated, Mel, melatonin-treated; T4I, CLI-095-treated; Veh, vehicle-treated.

Reg3β can be induced in goblet cells^29^, and melatonin promotes goblet cells and modulates cytokines. Moreover, since melatonin can modulate the function of macrophages^30^, HT-29 cells were treated with melatonin with or without THP-1 cells that were differentiated to macrophages by PMA stimulation and Alcian blue stain was performed to assess whether melatonin induced goblet cell differentiation *in vitro*. Melatonin treatment increased Alcian blue staining compared to the vehicle-treated group. However, there was no difference between HT-29 only (Fig. 5a) and co-culture with differentiated THP-1 cells (Fig. S4a). The increased Alcian blue stain was not detected when cells were treated with TLR4 inhibitor. Consistently, mRNA expression of *MUC2*, *MTNR1A*, and *MTNR1B* significantly increased after melatonin and LPS treatment, a finding that was eliminated when treated with TLR4 inhibitor (Fig. 5b,c). These results suggest that melatonin directly modulates goblet cell differentiation, which requires a melatonin receptor and the TLR4 signal pathway. In addition, melatonin increased mRNA expression of a proinflammatory cytokine (*TNFA*) and an anti-inflammatory cytokine (*IL10*) as well as melatonin receptors (*MTNR1A*, *MTNR1B*) in differentiated THP-1 cells (Fig. 5e,f).

Upregulated expression of *REG3G*, *CAMP*, and *DEFA3*, the human orthologues of *Reg3b*, *Cramp*, and *Defa3*, in HT-29 cells was observed after melatonin only treatment or melatonin co-treatment with LPS (Fig. 5d), although induction of *Cramp* and *Defa3* was not induced by melatonin *in vivo*. Consistently, we observed upregulated expression of *REG3G* in differentiated THP-1 cells (Fig. 5g). To confirm that melatonin-treated cells suppress growth of Gram-negative bacteria in the pathogenesis of colitis, culture media of HT-29 cells or HT-29 cells co-cultured with differentiated THP-1 cells were treated with *Escherichia coli*, a representative Gram-negative bacterium. Dots of colonies and optical density of *E. coli* culture revealed that both melatonin-treated HT-29 cells and co-culture with THP-1 cells inhibited growth of *E. coli*, which was not affected by co-culture with THP-1 cells (Figs. 5h,i and S4b). These results suggest that melatonin suppresses *Bacteroidetes* through Reg3β induction.

### Melatonin promotes wound healing in intestinal epithelial cells through TLR4 signalling

**Figure 6:**
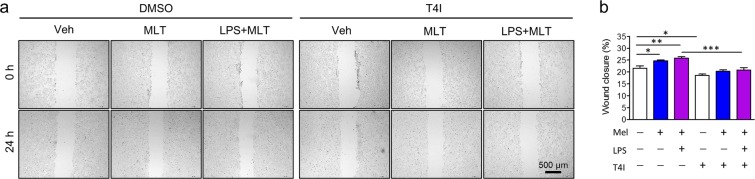
Melatonin improves wound healing of intestinal epithelial cells through TLR4 signal pathway. (**a**) Representative image of wound healing assay. (**b**) Wound closure rate. Wound closure rate was assessed by measuring wound area using Image J software. Data represent mean ± S.E.M. of 3 independent experiments. Statistical significance was assessed using one-way ANOVA followed by Dunnett post-test. *P < 0.05, **P < 0.01, ^##^*P* < 0.01 vs. DMSO. DMSO, dimethyl sulfoxide-treated; LPS, lipopolysaccharide-treated, Mel, melatonin-treated; T4I, TLR4 inhibitor-treated; Veh, vehicle-treated.

Previous studies have reported that TLR4 signalling promotes wound healing and induces AMPs in intestines^31^. Moreover, expression of melatonin receptor is upregulated by melatonin through TLR4 signalling. To assess whether melatonin affects intestinal barrier regulation through wound repair, a wound healing assay was performed using scraped HT-29 cells with or without differentiated THP-1 cells. Melatonin promoted wound closure compared to the control, which was further increased by LPS stimulation. However, there was no significant difference between HT-29 cells only (Fig. 6a,b) and HT-29 cells co-cultured with THP-1 cells (Fig. S4c,d), suggesting that melatonin directly affects wound healing of IECs.

## Discussion

Melatonin has versatile functions, including antioxidant, memory formation, and blood pressure reduction qualities^32^. Additionally, many previous studies showed that oral and rectal administration of melatonin suppresses DSS-induced colitis^33–35^. Melatonin is abundant in the gut^15^, and pinealectomy has no influence on gut melatonin concentrations^36^. Although it is widely accepted that IBD involves perturbation of homeostasis between the gut microbiota and the host immune system^37^, the effects of melatonin on dysbiosis in IBD remain unknown. It is not yet clear how melatonin shapes biological functions, although recent studies have suggested that melatonin alters the microbiome of the GI tract^10,17^. Herein, we showed that melatonin i.p. administration in WT mice alleviates DSS-induced colitis. As with the previously reported effect of melatonin on DSS- or TNBS-induced colitis^38,39^, melatonin suppresses potent pro-inflammatory mediators in colitis such as *Il1b* and *Il17a*, and controls microbiota in the intestine.

Consistent with our observation, many studies have reported that melatonin increases goblet cells in the intestine of vertebrates^10,17,39,40^. Goblet cells are important secretory IECs in colitis^19^ that produce mucin and AMPs. These products serve as the first lines of defence against pathogens by maintaining homeostasis of the mucus barrier and commensal bacteria and by segregating intestinal bacteria and the epithelium^41^. Considering that IECs interact with enteric bacteria, AMP production from IECs may affect the composition and abundance of the microbiota and play a role in protecting against bacterial invasion^42^, in addition to other types of cells such as T cell and innate lymphoid cells^43^. Although the route of administration was different from the previous study, we still identified indications that melatonin can reverse microbial dysbiosis in colitis and increase the *Firmicutes* to *Bacteroidetes* ratio. While melatonin has been reported to elevate the abundance of *Firmicutes* such as *Lactobacillus* and *Akkermania*, and reduce *Bacteroidetes* such as *Prevotellaceae*^10,17^, we did not observe any significant changes in these bacteria. This is likely due to different bacterial niches within the GI tract caused by the different sampling method. Given that mucin affects the composition of microbiota^29^ and some bacteria use glycan as a nutrient^44,45^, microbiota modulation by melatonin may be due to mucin regulation by goblet cell differentiation.

More importantly, host defences against Gram-negative bacteria such as *Salmonella* can be controlled by AMPs such as Reg3β and defensin alpha^46–48^. This may be another important mechanism of microbial control by melatonin. LPS in the cell walls of Gram-negative bacteria, pathogens commonly associated with IBD, induces an inflammatory response through TLR4^49^. TLR4 is receptor for bacterial LPS and regulates LPS-induced inflammatory response. Gram-negative bacteria abundantly increase in TLR4 KO mice, and TLR4 induces differentiation of goblet cells and AMP expression, including Reg3γ and Reg3β^42,50^. We found Gram-negative bacteria such as *Proteobacteria* are elevated in the feces of TLR4 KI mice (Fig. 3c), while *Firmicutes* such as *Ruminococcaceae* are reduced in those of TLR4 KI mice (Fig. S2d). As in the previous study^29,48^, we identified that Reg3β can be induced in proximal colon tissue. The previous study reported that melatonin inhibited infection in an enterotoxigenic *E. coli*-infected model^17^. Our study is the first to show that melatonin increased AMPs, especially Reg3β. Consistent with the antibacterial specificity of Reg3β for Gram-negative bacteria^47,51,52,46^, we observed dramatically increased Reg3β expression in mouse colons, IECs, and macrophages and suppressed growth of *E. coli* when IECs were treated with melatonin. Our results suggest that induction of AMPs by melatonin is a direct cause of Gram-negative bacterial suppression. Indeed, Reg3β deficient mice show abnormalities in inhibiting Gram-negative bacteria such as Salmonella^47,53^. AMPs are always expressed, but Reg3β is known to be expressed by TLR activation during microbial growth or inflammation^46,54^. Given normal physiologic conditions, the colon does not express Reg3β and α-defensin but these AMPs are induced in colitis^55^, suggesting that TLR4 signalling also plays a role in Reg3β regulation by melatonin. Although melatonin has been reported to inhibit TLR4/NF-κB signalling^56,57^, TLR4 signalling appears to link pathogen sensing and AMP production is thought to play protective role in melatonin function. Experiments with TLR4 KO mice and TLR4 inhibitor revealed the anti-colitic effects of melatonin on goblet cell differentiation and induction of melatonin receptor. We demonstrated previously that TLR4 signalling is required to goblet cell differentiation^58^.

Reg3β expression also was dependent on TLR4 signalling despite a contradictory study that melatonin suppressed related TLR4 signalling^56^. TLR4 mediates healing and proliferation of the injured intestinal epithelium^31,40^ and subsequently regulates intestinal barrier function and mucosal healing^59^. Consistently, we found increased wound healing of IECs with melatonin, and these effects require TLR4 signalling and are mediated through macrophages. We also observed synergetic expression of mucin, antimicrobial peptides, and melatonin receptors after melatonin treatment with LPS, which is likely due to increased expression of TLR4 by LPS. Our results are also consistent with those of a previous study that TLR4 KO mice are more susceptible to colitis than WT mice^60,61^. Nevertheless, there may be other pathways for melatonin function than TLR4, and further mechanical study is needed to explain these contradictory results of the interaction between melatonin and TLR4 signalling.

Although further study such as conventional knock-out system is also required to confirm the exact mechanisms of AMP induction by melatonin and to exclude many confounding factors, this is the first study to explain how melatonin regulates the microbiota in colitis and to show a link between melatonin and microbiota. Additionally, we showed that bacterial sensing through TLR4, mucin, and Reg3β production by goblet cells was involved in the anti-colitic effects of melatonin, suggesting that melatonin may be useful in microbiota control and therapeutics for IBD.

## Methods

### Mouse colitis model

TLR4-deficient BALB/c mice were provided by the Korea Research Institute of Bioscience and Biotechnology (Daejon, South Korea) and were bred as previously described^58^. To induce acute colitis, 8-9-week-old male BALB/c mice and TLR4 knockout mice were fed 2.5% (w/v) DSS (MW 36,000–50,000, MP Biomedicals, Solon, OH, USA) in their drinking water for 6 days. The first day of DSS administration was designated day 0; drinking water with DSS was replaced with normal drinking water on day 6. Mice were randomly divided into four groups: control, melatonin, DSS + vehicle, and DSS + melatonin. Control and melatonin group mice were administered only drinking water. Melatonin and DSS + melatonin groups received melatonin (10 mg/kg/day) by i.p. from days 1 to 8, while Control and DSS + vehicle groups were treated with 0.25% EtOH/PBS i.p based on our previous studies using DSS-induced colitis to detect rapid response^8,13,62^. All mice were sacrificed on day 9, and colon length was measured. Colons and cecal contents were collected for analysis. All mice were monitored daily for body weight loss, stool consistency, and rectal bleeding. Disease activity index (DAI) scores were evaluated before sacrifice. DAI score was calculated based on the following parameters: body weight loss (0, none; 1, 1–5%; 2, 5–10%; 3, 10–20%; 4, >20%), rectal bleeding (0, absence; 1 and 2, light bleeding; 3, bleeding), and stool consistency (0, negative; 1 and 2, loose; 3, diarrhoea). DAI scores were expressed as the average of these three parameters. All animals were kept under standard conditions at 21–22 °C under a 12-h light/dark cycle. A longitudinal incision was made along the entire colon, and all stool was eliminated by washing with PBS. Parts of the proximal colon were cut into 3 pieces for PAS staining and RNA isolation. All experiments using animals were reviewed and approved by the Institutional Animal Care and Use Committee (IACUC) of Yonsei University Severance Hospital, Seoul, Korea (IACUC Approval No: 2015-0407) and were conducted in accordance with the guidelines of the IACUC.

### Periodic acid-Schiff and Alcian blue staining, immunostaining, and histology

Four-µm-thick paraffin sections of the distal colon of each animal or of cells were stained with PAS stain or Alcian blue, respectively, according to standard procedures to evaluate the histopathology and goblet cells. Immunohistochemistry was performed using anti-mouse MTNR1A (1:500; Abcam, Cambridge, MA, USA), as previously described^58,63^. After incubation with anti-mouse Reg3β (1:200, R&D Systems, Minneapolis, MN, USA), sections were incubated with Alexa Fluor-488-conjugated secondary antibodies (Thermo Fisher Scientific, San Jose, CA, USA) for immunofluorescence stains. All images were obtained by confocal microscopy (Carl Zeiss LSM 700, Prenzlauer, Berlin, Germany), and staining intensity was determined using Image J software (NIH, Bethesda, MD, USA). Goblet cell staining was scored from 0 to 5 (5, minimal, <10%; 4, minimal, <20%; 3, mild, 21–30%; 2, moderate, 31–40%; 1, moderate, 41–50%; 0, marked, >50%).

### Cell culture and treatment

HT-29 (Korea Cell Line Bank, Seoul, South Korea) and THP-1 (ATCC, Manassas, VA, USA) cell lines were maintained in 10% foetal bovine serum-supplemented RPMI medium with or without antibiotics at 37 °C in a humidified atmosphere of 5% CO_2_ with or without melatonin (200 µM, Sigma-Aldrich, St. Louis, MO, USA), lipopolysaccharides (LPS, 200 ng/mL, Sigma-Aldrich, St. Louis, MO, USA), and TLR4 inhibitor CLI-095 (1 μg/mL, InvivoGen, San Diego, CA, USA) for the indicated times. THP‐1 cells were treated with a final 100 nM of phorbol 12‐myristate 13‐acetate (PMA, Sigma–Aldrich) to differentiate into macrophage‐like cells.

The indigenous *E. coli* strain previously isolated from mouse^64^ was kindly provided by Yoon. Bacterial culture incubated with HT-29 cell culture media was plated onto nutrient agar or EMB agar or cultured in nutrient broth at 37 °C for 48 h under static culture conditions. *E. coli* colony images were captured by a Gel document system (MINIBIS PRO, Jerusalem, Israel), and the optical density (OD_600_) of *E. coli* culture was measured using ELISA reader VERSA Max (Molecular Devices, Sunnyvale, CA, USA) and analysed using SoftMax Pro ver.6.3 (Molecular Devices).

### Real-time quantitative reverse-transcription polymerase chain reaction (RT-PCR)

Total RNA was prepared from colon tissues and cells using a Ribospin kit (Geneall, Seoul, Korea) and TRIzol Reagent (Life Technologies, Carlsbad, CA, USA), respectively, as per the manufacturer’s instructions. RNA was reverse-transcribed using the High Capacity cDNA Reverse Transcription Kit (Applied Biosystems, Foster City, CA). The mRNA expression levels were quantified using a StepOne Plus real-time PCR system (Applied Biosystems) and SYBR Green Master Mix (Applied Biosystems). Thermal cycles were: 45 cycles of 95 °C for 30 s, 60–63 °C for 30 s, and 72 °C for 40 s. All PCRs were run in duplicate. The results were normalized to expression of the β-actin gene, and the relative expression of each target mRNA was calculated as 2^−ΔCt^. Results were normalized to expression of β-actin and were calculated by the equation 2^−ΔΔCt^. Primer sequences were synthesized by Macrogen (Seoul, Korea). PCR primers are listed in Table S1.

### Metagenome analysis of microbiome

Bacterial genomic DNA was obtained from faeces using a FastDNA™ SPIN for Soil Kit (MP Biomedicals, Santa Ana, CA, USA) according to the manufacturer’s instructions. Microbiome detection using bar-coded primers flanking the V3-V4 region of the 16S rRNA gene was completed by ChunLab Inc. (Seoul, Korea). Operational taxonomic units (OTUs) were identified based on 16 S rRNA sequence data using the EzTaxon-e database (http://eztaxon-e.ezbiocloud. net). Principal coordinate analysis (PCoA) of gut microbiome composition was assessed by Jensen-Shannon. OTU richness (abundance-based coverage estimator (ACE) and Chao1 indices) and diversity (Shannon index and Simpson index) were analysed for alpha diversity analysis. Bacterial community abundance and composition were analysed using CLcommunity software (Chunlab Inc.).

### Western blot analysis

Colon tissues were lysis harvested on ice by washing twice with cold PBS, scraping, and resuspending. The protein concentration of colon lysate was determined using the BCA assay with bovine serum albumin. Protein samples prepared by mixing SDS sample buffer were run on NuPAGE 4–12% gradient Bis-Tris and electrotransferred to a polyvinylidene difluoride membrane (Thermo Fisher Scientific) as in a previous study.64 Blots were blocked with 5% (w/v) skim milk in Tris-buffered saline solution containing 0.1% Tween 20 (Pierce, Appleton, WI, USA) and incubated overnight at 4 °C with antibodies against Reg3β (1:1000, R&D Systems) and β-actin (Sigma). Blots were additionally incubated with secondary antibodies conjugated with horseradish peroxidase for 1 hour at room temperature and visualized with ELC Prime detection reagent and the LAS 4000 Mini apparatus (GE Healthcare, Piscataway, NJ, USA).

### Statistical analysis

GraphPad Software (La Jolla, CA, USA) was used for statistical analyses. The significance of differences between conditions was assessed using Student’s *t*-test or one-way analysis of variance (ANOVA). *P* values < 0.05 were considered significant.

## Supplementary information

Supplementary information.
