# Supplementary material for: Melatonin controls microbiota in colitis by goblet cell differentiation and antimicrobial peptide production through Toll-like receptor 4 signalling
Source: Sci Rep. 2020 Feb 10;10:2232. doi: 10.1038/s41598-020-59314-7 (PMC7010660; doi:10.1038/s41598-020-59314-7)
Supplement: Supplementary file 1 — Supplementary information. [file 41598_2020_59314_MOESM1_ESM.pdf]

Title: Melatonin controls microbiota in colitis through Toll-like receptor 4 signalling

Seung Won Kim,[1,2,3] Soochan Kim,[1,3] Mijeong Son,[1,2,3] Jae Hee Cheon,[1,2,3] and Young Sook Park[4]

[1]Department of Internal Medicine and Institute of Gastroenterology, Yonsei University College of Medicine, Seoul, Korea

[2]Brain Korea 21 PLUS Project for Medical Science, Yonsei University College of Medicine, Seoul, Korea

[3]Severance Biomedical Science Institute, Yonsei University College of Medicine, Seoul, Korea

[4]Department of Internal Medicine, Eulji Hospital, Eulji University School of Medicine, Seoul, Korea

Supporting Information

Supplementary Figures

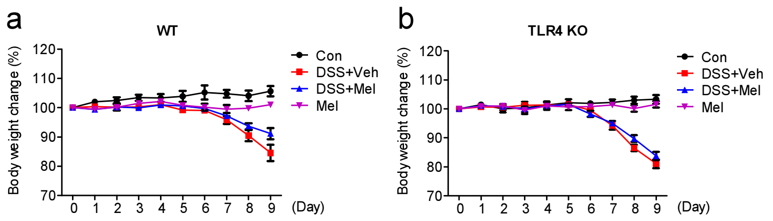

Fig. S1. Body weight changes**.** Wild type (**a**) and TLR4 knockout (**b**) mice were administered 2.5% DSS in drinking water and treated i.p. with 0.25% EtOH/PBS (Veh) or melatonin (Mel, 10 mg/kg/day) from days 1 to 8. Con, untreated control; Mel, melatonin-treated; TLR4 KO, TLR4 knockout mice; Veh, vehicle-treated; WT, wild-type mice.

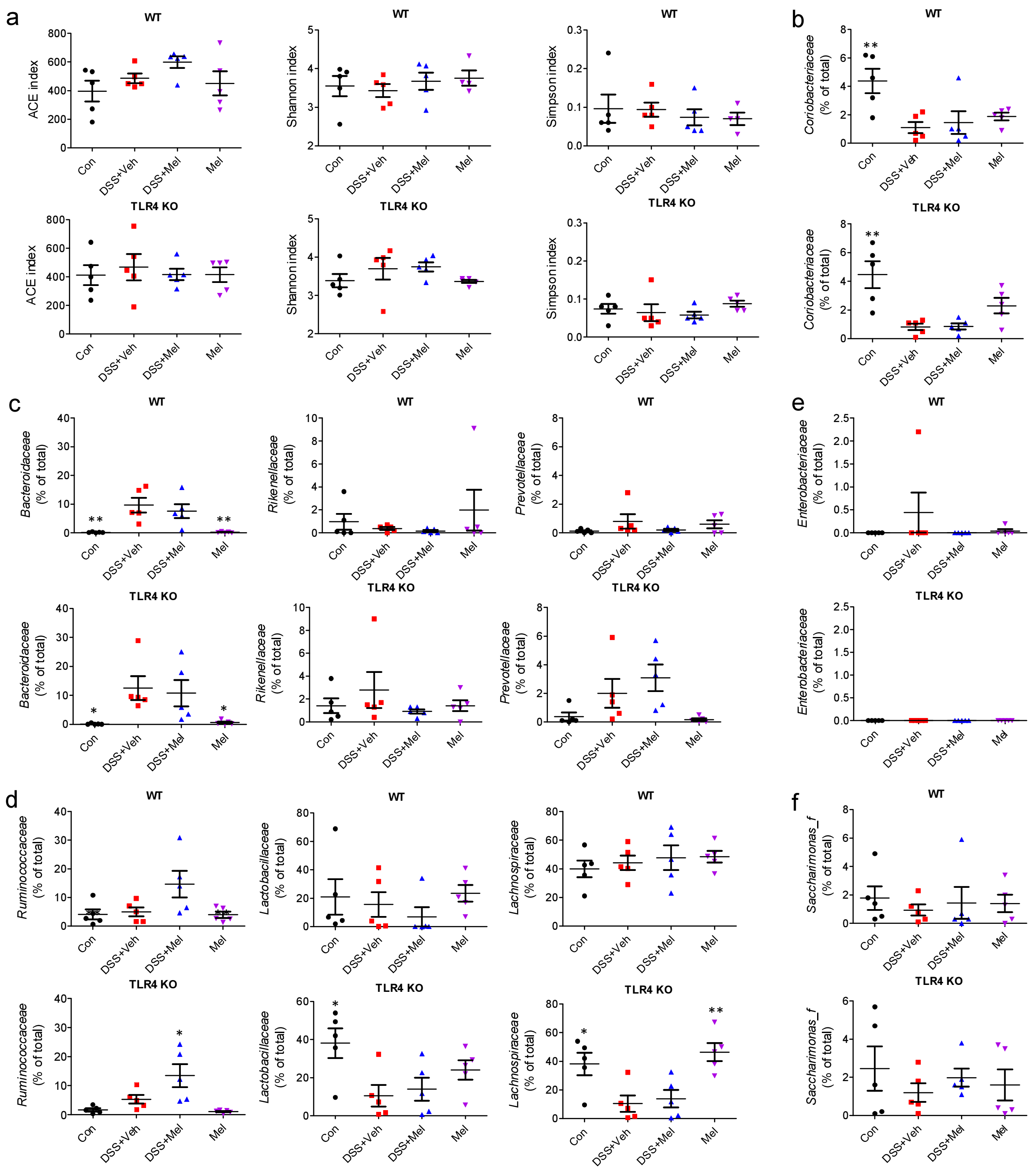

Fig. S2. Melatonin suppresses dysbiosis of gut microbiome through TLR4 signal pathway. Fecal microibiome composition in colons of wild type and TLR4 knockout mice was generated using 16S rRNA sequencing. (**a**) Richness and diversity were predicted by ACE, Shannon index, and Simpson index. (**b–f**) Microbiota profiles in family level belong to phylum *Actinobacteria* (**b**), *Bacteroidetes* (**c**), *Firmicutes* (**d**), *Proteobacteria* (**e**), and *Saccharibacteria TM7* (**f**). Data represent mean ± S.E.M. (n = 5). **P* < 0.05, ***P* < 0.01. Con, untreated control; Mel, melatonin-treated; TLR4 KO, TLR4 knockout mice; Veh, vehicle-treated; WT, wild-type mice.

1st animal set (1)

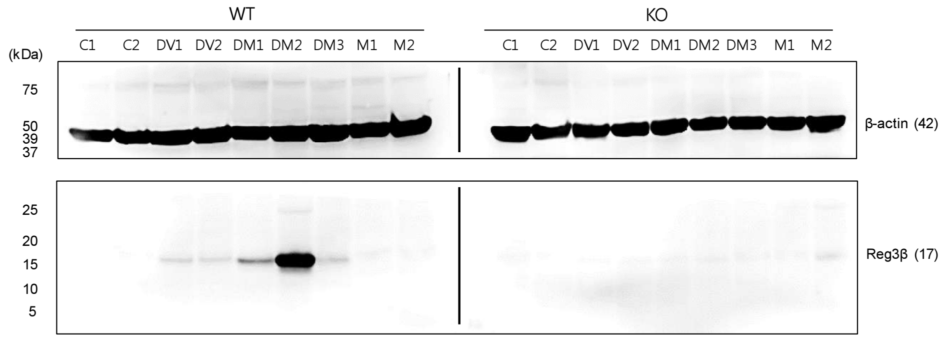

1st animal set (2)

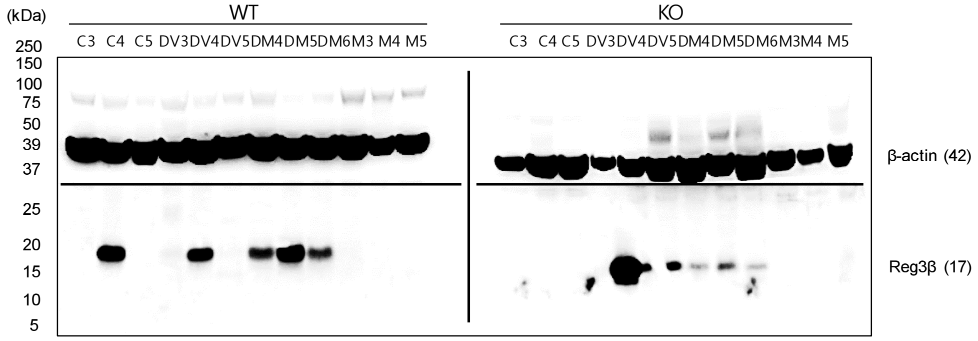

2nd animal set

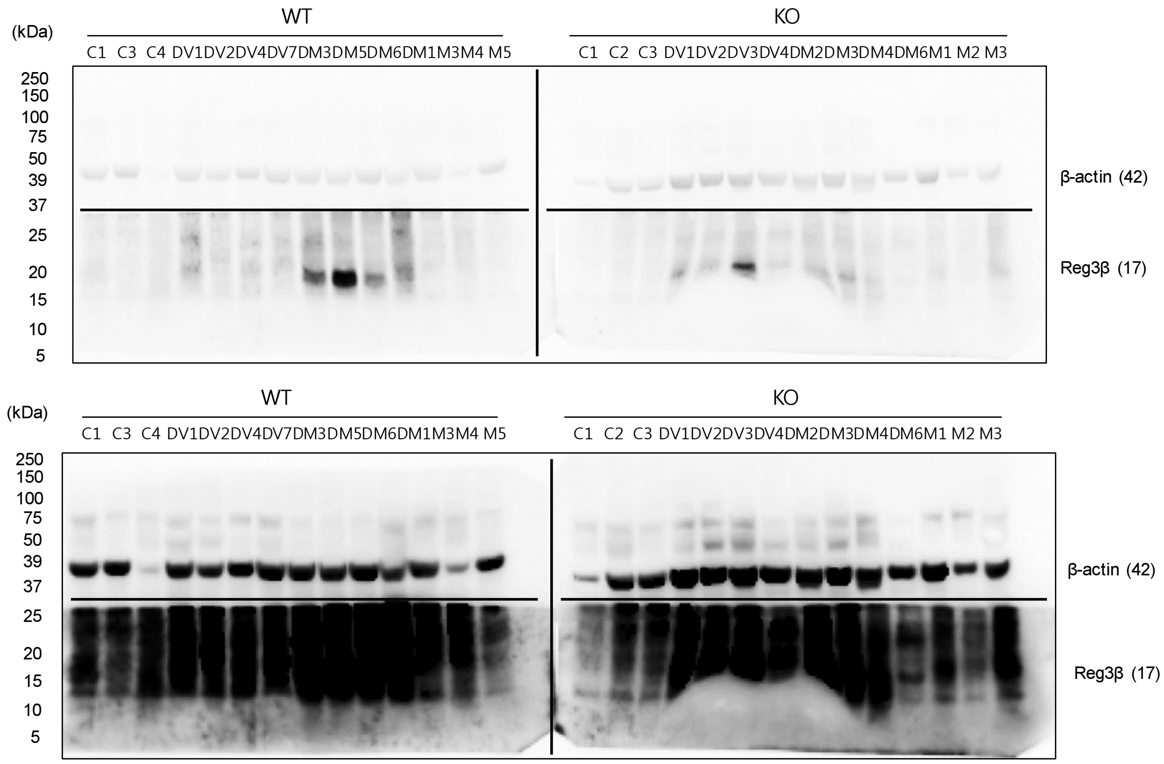

Fig. S3. Full image of representative western blot for Reg3β in colon lysates of WT and TLR4 KO mice.

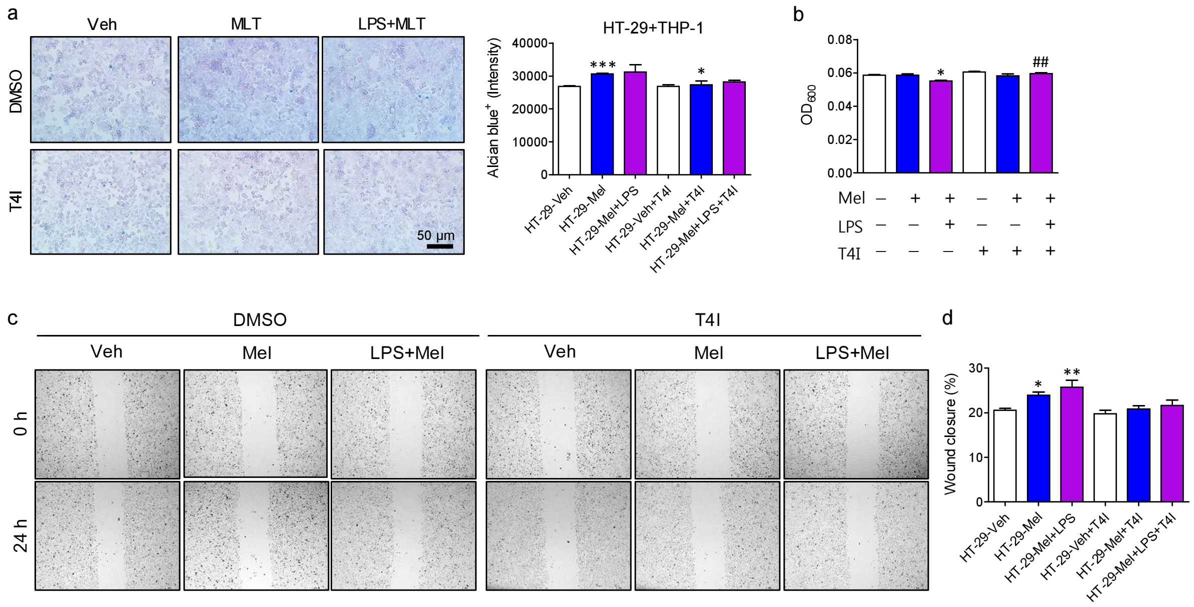

Fig. S4. Melatonin controls Gram-negative bacteria by goblet cell differentiation and antimicrobial peptide production through TLR4 signal pathway independently of macrophage cells. HT-29 cells were co-cultured with differentiated THP-1 cells and treated with vehicle (Veh) or melatonin (Mel) with or without TLR4 inhibitor (T4I) for 48 h. (**a**) Representative image of Alcian blue stain (left) and densitometry analysis (right). Data represent means ± S.E.M. of 2 independent experiments. (**b**) Antimicrobial activity of melatonin-treated cells. *Escherichia coli* (OD_{600} = 0.5) grown in LB broth were treated with culture media from HT-29 cells, which were co-cultured with THP-1 cells and incubated for 48 h. Optical density (OD_{600}) of *E. coli* culture was measured. Data represent means ± S.E.M. of 4 independent experiments. (**c**) Representative image of wound healing assay. (**d**) Wound closure rate. Wound closure rate was assessed by measuring wound area using Image J. Data represent mean ± S.E.M. of 2 independent experiments. $^{*}P < 0.05$, $^{**}P < 0.01$, $^{**}P < 0.01$, $^{***}P < 0.005$, $^{\#\#}P < 0.01$ vs. DMSO. DMSO, dimethyl sulfoxide-treated; LPS, lipopolysaccharide-treated, Mel, melatonin-treated; T4I, TLR4 inhibitor-treated; Veh, vehicle-treated.

Supplementary Table

Table S1. List of primers used for qRT-PCR

Gene	Sequence (5'–3')
**Human**	
MUC2	F: AGGATGACACCATCTACCTCACC<br>R: GGTGTAGGCATCGCTCTTCTC
MTNR1A	F: TGTCGATATTTAACAACGGGTGG<br>R: CGATGCCGGTGATGTTGAA
MTNR1B	F: GCATGGCCTACCACCGAATC<br>R: AATAGATGCGTGGGTCGTACT
REG3G	F: CTCCCTGGTGAGGAGCATTA<br>R: GCAGACATAGGGTAACTTTGC
CAMP	F: AGGATTGTGACTTCAAGAAGGACG<br>R: GTTTATTTCTCAGAGCCCAGAAGC
DEFA3	F: CATGGGACGAAAGCTTGGCT<br>R: TGCAGGTTCCATAGCGACGTT
β-ACTIN	F: CTCTTCCAGCCTTCCTTCCTG<br>R: CAGCACTGTGTTGGCGTACAG
**Mouse**	
β-actin	F: CATCTTCACCGTTCCAGT<br>R: GTCCACCTTCCAGCAGAT
Il1b	F: GCAACTGTTCCTGAACTCAACT<br>R: ATCTTTTGGGGTCCGTCAACT
Tnfa	F: CAAAGGGAGAGTGGTCAGGT<br>R: ATTGCACCTCAGGGAAGAGT
Il22	F: GGCCAGCCTTGCAGATAACA<br>R: GCTGATGTGACAGGAGCTGA
Cramp	F: GCACGCTGACACCACTACC<br>R: CGGGCTATTCCCTGTCCAC
Il10	F: GCCACATGCTCCTAGAGCTG<br>R: CAGCTGGTCCTTTGTTTGAAA

Il17A	F: CAGGACGCGCAAACATGA<br>R: GCAACAGCATCAGAGACACAGAT
Mtnr1a	F: TGTCAGCGAGCTGCTCAATG<br>R: GGTACACAGACAGGATGACCA
Mtnr1b	F: GAACAGCTCAATCCCTAACTGC<br>R: ACGACTACTGTAGATAGCATGGG
Reg3b	F: TGGGAATGGAGTAACAATG<br>R: GGCAACTTCACCTCACAT
Defa3	F: TCCTCCTCTCTGCCCTYGTCCTG<br>R: AGACACAGCCTGGTCSTCTTCC
Defa4	F: CACCGCAATCGAAGGCTTG<br>R: ACAGTAGGCAATCCATACCCAC

F: forward primer, **R**: reverse primer
